# A Short-Pulse Indirect ToF Imager Using Six-Tap Pixel with a Backside-Illuminated Structure for High-Speed Demodulation

**DOI:** 10.3390/s25247581

**Published:** 2025-12-13

**Authors:** Tomohiro Okuyama, Haruya Sugimura, Gabriel Alcade, Seiya Ageishi, Hyeun Woo Kwen, De Xing Lioe, Kamel Mars, Keita Yasutomi, Keiichiro Kagawa, Shoji Kawahito

**Affiliations:** 1 Graduate School of Medical Photonics, Shizuoka University, Hamamatsu 432-8011, Japan; 2 Graduate School of Integrated Science and Technology, Shizuoka University, Hamamatsu 432-8011, Japan; 3 Graduate School of Science and Technology, Shizuoka University, Hamamatsu 432-8561, Japan; 4 Research Institute of Electronics, Shizuoka University, Hamamatsu 432-8011, Japan; 5 SUiCTE Co., Ltd., Hamamatsu 432-8011, Japan; 6 Faculty of Science and Technology, Shizuoka Institute of Science and Technology, Fukuroi437-8555, Japan

**Keywords:** iToF, BSI, depth sensing, short pulse, demodulation contrast

## Abstract

This paper evaluates the effectiveness of a backside illumination (BSI) structure in a short-pulse indirect time-of-flight (SP-iToF) sensor employing 6-tap pixels. Impulse response measurements comparing 6-tap iToF pixels fabricated with both front-side illumination (FSI) and BSI structures demonstrate that the BSI configuration yields a significantly faster response to near-infrared (NIR) light at 850 nm. Specifically, the time constants near the response peak and tail are 0.35 ns and 0.93 ns, respectively—approximately half those observed in the FSI counterpart. Demodulation contrast (DC) measurements further highlight the advantages of the BSI structure. The BSI pixel achieves a DC of 99.5% with a gating pulse width (PW) of 10 ns, which decreases only slightly to 95.3% at a PW of 3 ns. In contrast, the FSI pixel shows a DC of 97.0% at 10 ns, but drops markedly to 80.0% at 3 ns. These improvements are primarily attributed to the thinner substrate used in the BSI sensor. The implemented 6-tap ToF sensor exhibits excellent depth linearity (<±0.8% full scale) and high resolution (<2%) across an indoor measurement range of 3 to 28 m.

## 1. Introduction

There has been a significant shift in the design of indirect time-of-flight (iToF) image sensors from front side illumination (FSI) [1,2,3,4,5,6] to back side illumination (BSI) [7,8,9,10,11,12,13,14]. This transition has been driven by the need to improve the signal-to-noise ratio (SNR) and increase the sensing performance of ToF sensors, particularly in outdoor environments where background light (e.g., sunlight) can introduce significant noise.

ToF cameras for outdoor environments typically operate near 940 nm, as this wavelength minimizes interference from sunlight. This choice is prevalent for both FSI and BSI sensors. To achieve high quantum efficiency (QE) at such long near-infrared (NIR) wavelengths, a long absorption path length is generally required.

In FSI, a common solution is to increase the thickness of the epitaxial layer to extend the optical path and enhance QE. However, this shift places photogeneration deeper in the substrate and lengthens carrier transit paths. As a result, a portion of the photogenerated charge reaches the collection node after the intended gating window (hereafter, “late-arriving charge”), which degrades demodulation contrast and depth-measurement linearity unless carrier transport and gating are sufficiently fast. A trade-off between maximizing NIR QE and maintaining demodulation contrast (DC) is therefore inherent to FSI pixel design, and the device structure and bias conditions must be carefully optimized [15,16].

BSI image sensors, on the other hand, use reflective metallization [17] and surface structures such as pyramids structure for diffraction [9,18] and deep trench isolation (DTI) to achieve a longer optical path length and improve NIR QE. In addition, a thinner substrate shortens the carrier transit distance and can improve DC. As a result, BSI iToF sensors alleviate the QE–DC trade-off observed in FSI, which leads to improved range performance of linearity and depth resolution.

This paper compares the dynamic pixel response of six-tap iToF image sensors fabricated in FSI and BSI processes that share an identical pixel layout. Sensor responses are characterized under two pulsed-illumination conditions with full width at half maximum (FWHM) of 15 ns and 70 ps (typical; unit label 69 ps). The Measurement results show that the BSI sensor exhibits higher demodulation contrast and two-times better time constants in the dynamic response characteristics than those of the FSI sensor. Using the implemented BSI six-tap sensor and a short-pulse (SP) iToF-based hybrid type of operation [12,19,20,21], a good linearity and depth resolution are measured over the range of 3–28 m using a two-subframe operation. Under the same pulse-width condition, a four-tap sensor would require three subframes to cover a similar range, resulting in a lower achievable frame rate than the six-tap configuration. Compared to the previous report of the BSI 4-tap sensor [12], measurements at a range of 30 m are achieved at a higher frame rate of 30 fps.

## 2. Six-Tap Pixel Design and Short-Pulse iToF Operation

### 2.1. Pixel Layout and Readout

**Figure 1:**
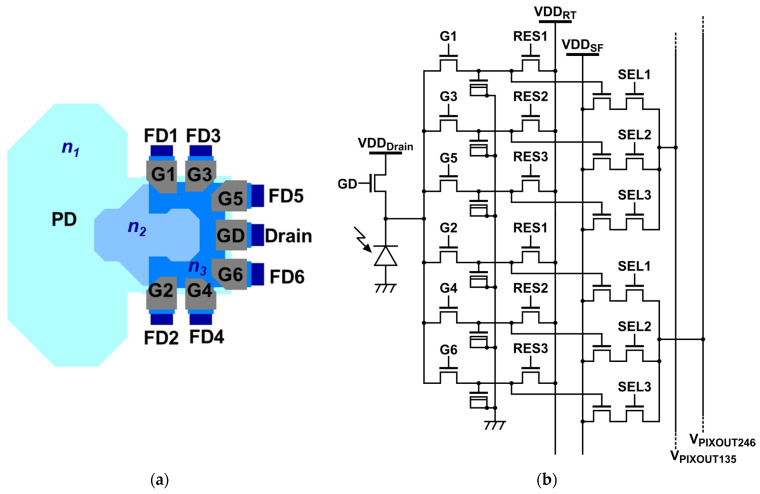
Six-tap iToF pixel. (**a**) Layout (pixel pitch 8.4 µm × 8.4 µm). (**b**) Circuit with short-pulse gating and two-tap parallel readout.

Figure 1a illustrates the pixel structure common to both the BSI and FSI sensors. Each pixel comprises a photodiode (PD), six gates (G1–G6), a drain gate (GD) and six floating diffusions (FD1–FD6). The devices were fabricated using a 0.11 µm CIS process with an array size of 1080 (H) × 488 (V) and a pixel pitch of 8.4 µm × 8.4 µm. Photogenerated electrons in the PD are driven by a built-in drift field and, via time-gated control of G1–G6, are steered to the FD corresponding to each time window.

In previously reported BSI four-tap iToF pixels [12], the demodulation gates are arranged in a ring around the photodiode. This layout works well for four taps, but if we try to add more taps with the same ring structure, the pixel must be made larger and the carrier-transfer paths become longer, which makes it harder to achieve high-speed charge transfer [20]. In the proposed six-tap pixel, the demodulation gates are instead placed along one side of the photodiode. Photoelectrons generated in the photodiode are guided laterally toward the demodulation region by drift fields formed by the stepped n-type regions (n1, n2, and n3), and are then directed to the corresponding floating diffusion nodes (FD1–FD6) through the gating action of G1–G6. This one-sided multi-tap configuration enables six-tap operation within a compact pixel layout while maintaining charge-transfer efficiency.

Figure 1b shows the six-tap circuit with two output lines. Six short-pulse clocks drive G1–G6 and an additional clock drives GD. Tap-output selection signals (SEL1–SEL3) multiplex two taps simultaneously onto two column lines, enabling parallel readout. The electrical transitions of the gate signals were evaluated using circuit simulations that included wiring resistance and estimated parasitic capacitances. For the pixel farthest from the driver, the simulated 10–90% rise and fall times of the gate voltage are 776 ps and 805 ps, respectively. The FSI sensor uses a 20 µm epitaxial wafer, whereas the BSI sensor uses a thinned 5 µm substrate. Both use a −3 V backside bias. Except for the illumination configuration (BSI vs. FSI), the pixel layout and readout are the same. At a wavelength of 940 nm, the quantum efficiency (QE) averaged over taps G1–G6 is 19.7% for the FSI pixel and 20.6% for the BSI pixel.

**Figure 2:**
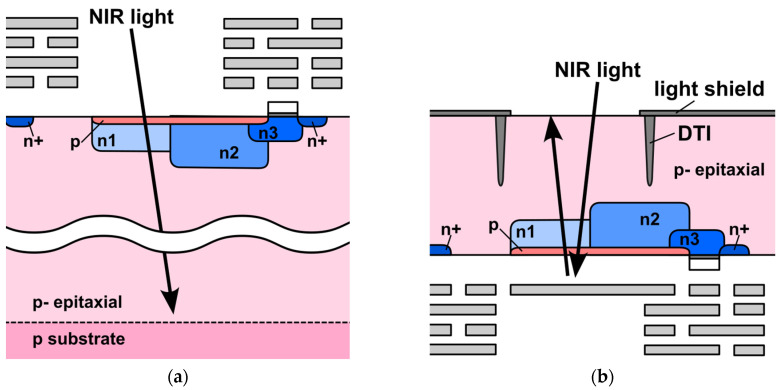
Simplified cross-sectional views of the (**a**) FSI and (**b**) BSI six-tap pixels. In the BSI pixel, a light shield and DTI are formed around the photodiode, and a metal reflector in the interconnect layer reflects incident NIR light back into the p-epitaxial layer, effectively lengthening the optical path and suppressing optical crosstalk.

In the BSI version, we modify the metal interconnect layer by introducing a reflector above the photodiode [17]. Figure 2a,b show simplified cross-sectional views of the FSI and BSI pixels, respectively. In the BSI structure, a light shield and deep trench isolation (DTI) are introduced around the photodiode to suppress optical crosstalk both between neighboring pixels and among the six taps within each pixel. In addition, the metal interconnect layer acts as a reflector that sends incident NIR light back into the p-epitaxial region, effectively lengthening the optical path.

### 2.2. Charge-Transfer Simulations

**Figure 3:**
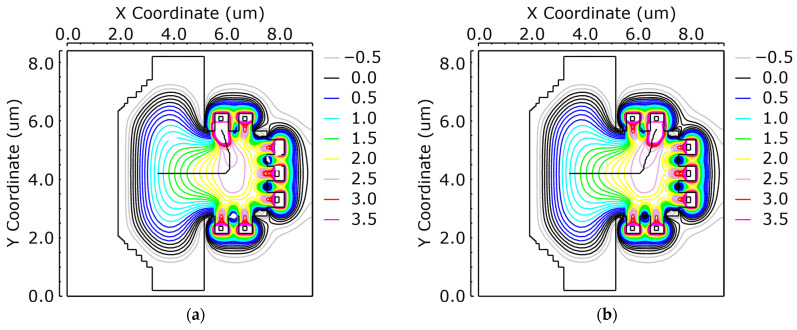

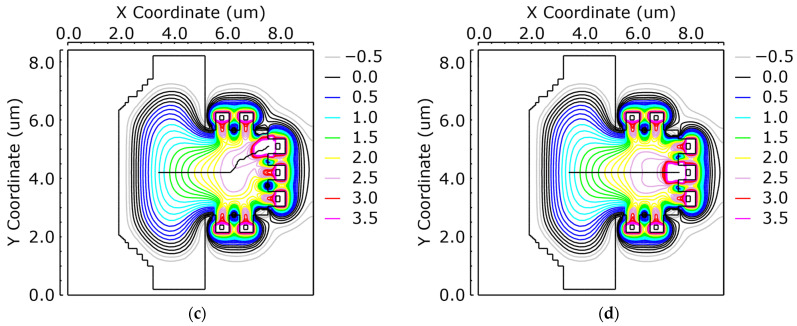
Device-level simulations of charge routing in a 6-tap iToF pixel. The potential contours and drift streamlines are shown for the following four cases: (**a**) G1 High, (**b**) G3 High, (**c**) G5 High (with the others set to Low and GD set to Low), and (**d**) GD High (with the drain set to High). Electrons are initialized near the PD center at depths of 5 µm and 10 µm.

Device-level simulations were conducted to validate charge routing in the six-tap iToF pixel and to estimate carrier transfer times to the FDs. A FSI sensor is used for this simulation to know the response of carriers coming to the surface from deep inside of silicon. Owing to the mirror-symmetric layout, four gate-on configurations were examined: G1 High, G3 High, G5 High (others Low), and GD High. In the first three cases, carriers were directed to the corresponding FD. With GD High, carriers were routed to the drain node. The routing paths are shown in Figure 3a–d, and the transfer times are summarized in Table 1.

Electrons were initialized near the geometric center of the photodiode at two starting depths (5 and 10 µm). The surface of the substrate was held at 0 V and the backside was held at −3 V.

Increasing the initial electron depth from 5 to 10 µm added approximately 111 ps across all cases. Longer transfer times result in poorer demodulation contrast because a larger proportion of carriers arrive outside the intended gating window. These findings support the use of a backside-illuminated (BSI) architecture with a thinned substrate in order to suppress deep-generation events. This approach enables faster effective demodulation and improves ranging performance.

**Figure 4:**
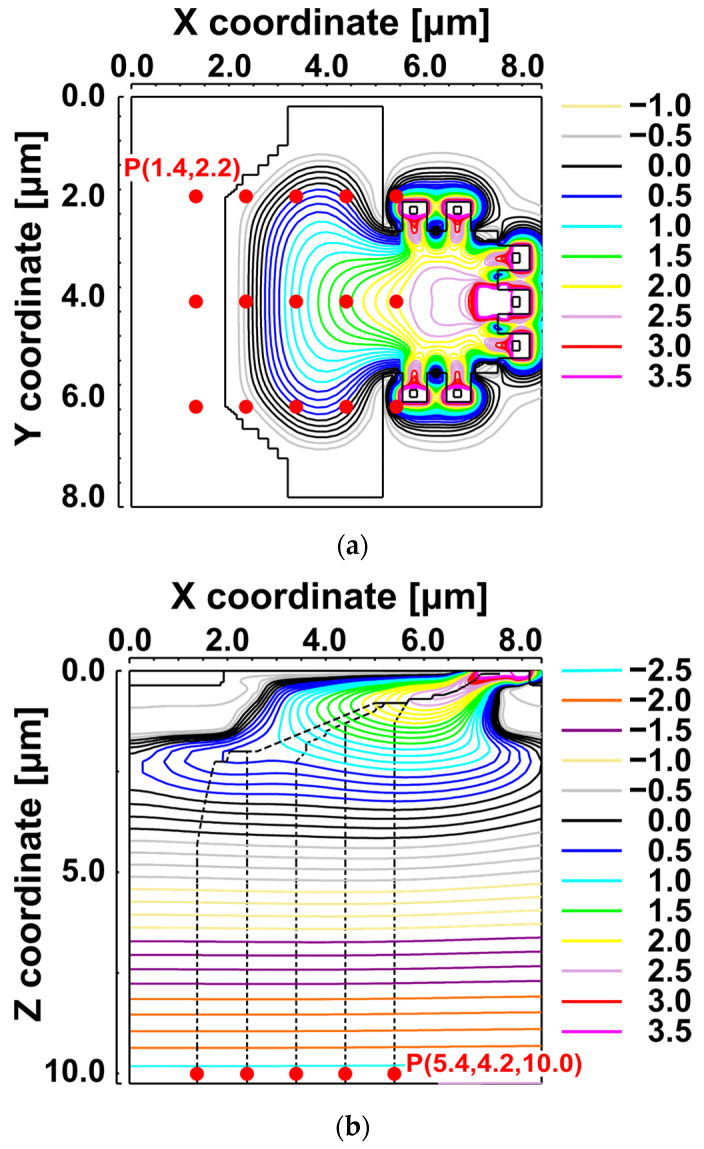
Simulated electrostatic potential in the FSI pixel with GD High and G1–G6 Low. (**a**) x-y potential distribution; red circles mark 15 starting points P(x, y, z) for transfer-time simulations to GD. (**b**) x-z potential distribution along y = 4.2 µm and transfer paths to GD from the five marked points.

Device simulations were performed for the FSI pixel with GD High and all demodulation gates (G1–G6) Low. Figure 4a shows the simulated electrostatic potential in the x-y plane. Fifteen starting positions (P(x, y, z)) are marked by red circles to represent photogeneration sites within the photodiode. Figure 4b illustrates the x-z potential distribution at y = 4.2 µm and the transfer paths from five selected initial positions toward the GD.

Table 2 summarizes the resulting transfer times to the GD for initial depths of z = 5 µm and z = 10 µm. Electrons generated near the lateral edges take approximately 1.1–1.3 ns to reach the GD. As shown in Figure 4b, carriers generated deeper travel almost vertically toward the channel before moving laterally toward the GD. Therefore, larger depths and lateral distances lead to longer Δt values.

### 2.3. Two-Subframe Short-Pulse Gating

**Figure 5:**
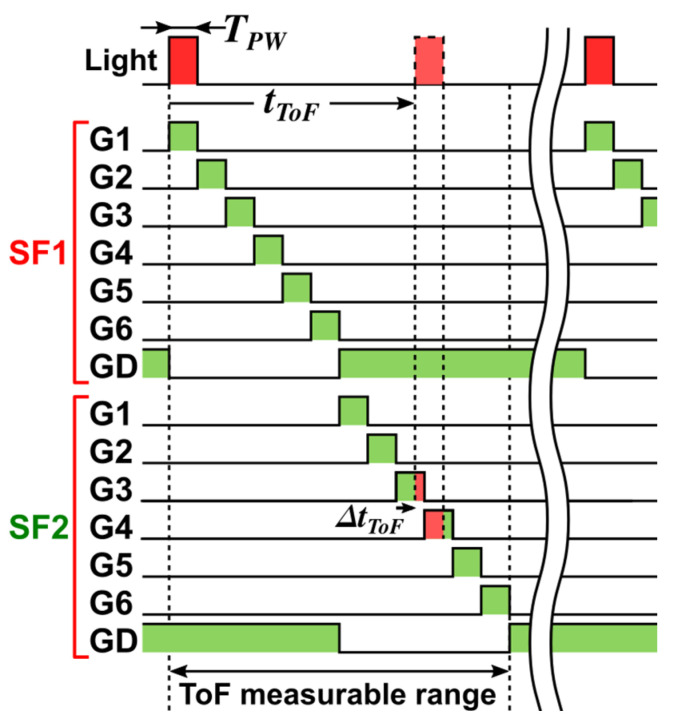
Two-subframe short-pulse gating for a 6-tap iToF pixel.

Figure 5 illustrates the two-subframe (SF1/SF2) gating scheme of the six-tap demodulator, which is synchronized to the short-pulse light source. In each subframe, the transfer gates (G1–G6) are sequentially asserted to define six time windows. After G6 is de-asserted, GD is asserted until the next G1, thereby draining the charge generated by background light and preventing its accumulation.

In multi-subframe operation, the measurable ToF range is extended by applying a range shift in each subframe with only minor changes to the gate-timing pattern. Increasing the number of subframes further extends the range but also increases the number of signal-accumulation and readout cycles required to form a single depth frame. To maintain the frame rate, faster readout circuits are needed, so the number of subframes and the readout architecture should be chosen by balancing measurable range against power consumption and frame-rate requirements.

In this scheme, each gate (G1–G6) defines a coarse time bin of width $T_{PW}$, so the coarse ToF estimate is obtained by identifying which gate captures the pulse and in which subframe (SF1 or SF2) it occurs. For the timing shown in Figure 5, the pulse lies between gates G3 and G4 in SF2, corresponding to a coarse index of 8. A fine ToF estimate is then obtained from the residual delay ${\Delta t}_{ToF}$ within that bin by interpolating the adjacent tap outputs of G3 and G4 ($Q_{3}$ and $Q_{4}$, respectively), giving a resolution finer than $T_{PW}$. The time-of-flight is then given by

$$t_{TOF}=8T_{PW}+\frac{Q_{4}}{Q_{3}+Q_{4}}T_{PW}.$$

## 3. Measurement Results

### 3.1. Delay-Sweep Measurement

**Figure 6:**
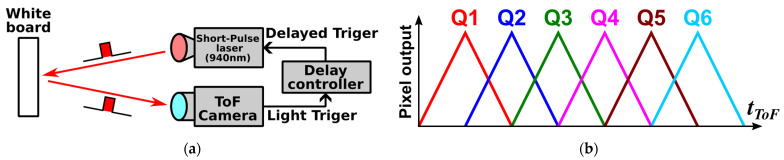
Delay-sweep measurement with a finite pulse. (**a**) Setup: 940 nm laser, 15 ns pulse width, 1 ns delay steps, 300 ns period, 940 ± 10 nm filter, White diffuse reflectance target. (**b**) Ideal six-tap response versus delay.

The output response of the six-tap pixel to a delayed short-pulse illumination was measured as in Figure 6a. For each delay setting, the tap outputs were recorded while the delay controller shifted the arrival time of the light at the pixel. The target was a white board made of a diffuse-reflectance material (Labsphere SRT-99-120, North Sutton, NH, USA; nominal reflectance 99%). A 940 nm laser with a pulse width of 15 ns was used. A wavelength of 940 nm was chosen for the ToF measurements because it is widely used in outdoor ToF systems and is less affected by ambient sunlight. Therefore, 940 nm is used as the system wavelength for ToF range measurements in this work. The laser trigger was generated by the ToF sensor and routed through a delay controller to shift the emission timing in 1 ns steps. The pulse period was 300 ns. A 940 ± 10 nm band-pass filter was placed in front of the camera. By changing the trigger delay, various time-of-flight conditions were emulated without moving the target. The gate step was set equal to the pulse width (15 ns). All plotted data were obtained by averaging over a 10 × 10 pixel region near the center of the array.

**Figure 7:**
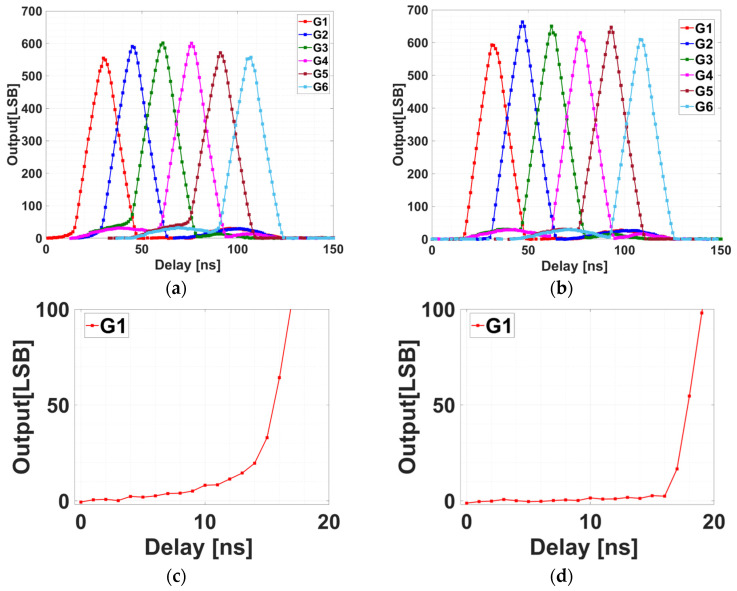
Delay-sweep responses using a 940 nm, 15 ns pulse with a period of 300 ns. (**a**) FSI sensor: triangular per-tap responses (G1–G6) versus delay. (**b**) BSI sensor: reduced late-arriving charge and steeper edges than the FSI sensor. (**c**) A close-up of the rising edge at G1 (FSI). (**d**) A close-up of the rising edge at G1 (BSI).

Figure 7a,b show the measured delay-response curves for the FSI and BSI sensors, respectively. Both devices exhibit the expected triangular responses of Q1–Q6. The BSI sensor shows lower residual tails, indicating higher modulation contrast and improved sensitivity. Figure 7c,d show close-ups of the rising edge of G1 for the FSI and BSI sensors, respectively. The FSI response rises more slowly than the BSI response. This suggests faster effective demodulation in the BSI sensor, consistent with reduced charge arrival outside the intended gating window.

### 3.2. Impulse-Response Measurement

Response measurements using a 15 ns pulse width—identical to that employed in the implemented ToF sensor—are useful for qualitatively assessing pixel response performance based on the shape of the response curve. However, this approach is limited in its ability to quantitatively evaluate the intrinsic response characteristics and their impact on ToF measurement accuracy and precision, particularly through precise demodulation contrast analysis. To address this limitation, the demodulation contrast and intrinsic response behavior of the 6-tap demodulator were evaluated by measuring the impulse response of each tap under ultra-short optical excitation. The pixel gates were driven with 10 ns pulses at a period of 300 ns.

The impulse-response measurements used a picosecond laser source (PLP-10, Hamamatsu Photonics, Hamamatsu, Japan) operating at 850 nm with a typical full width at half maximum (FWHM) of 70 ps. In our experimental setup, this was the only available picosecond NIR source, so 940 nm could not be used for the impulse-response characterization. For this BSI sensor, the gate-averaged quantum efficiency at 850 nm (40.6%) is higher than at 940 nm (20.6%), which makes 850 nm attractive for indoor, high-resolution applications where strong ambient sunlight is not a concern. At the same time, NIR light at 850 nm has a long absorption length in silicon [22,23,24] and, even in the 20 μm-thick epitaxial layer used in the FSI pixel, generates photoelectrons deep inside the epitaxial region. Thus, the 850 nm impulse-response measurements provide meaningful information on carrier-transport dynamics, including late-arriving charge, that is also relevant to 940 nm operation. As described in Section 3.1, a wavelength of 940 nm is used for ToF range measurements because it is less affected by ambient sunlight.

The optical waveform was characterized using a photodetector (DX30BF, Thorlabs, Newton, NJ, USA) and a sampling module (86112A, Agilent Technologies, Santa Clara, CA, USA). The photodetector provides a 30 GHz bandwidth, ensuring a response much faster than the 70 ps FWHM optical pulse. Although the light pulse with a FWHM of 70 ps is not sufficiently narrow to be considered a true impulse excitation, the sensor’s intrinsic response can be accurately estimated through deconvolution, provided the waveform of the light pulse is known. Accordingly, the recorded outputs were deconvolved using a Wiener filter to obtain the sensor’s impulse response function (IRF).

**Figure 8:**
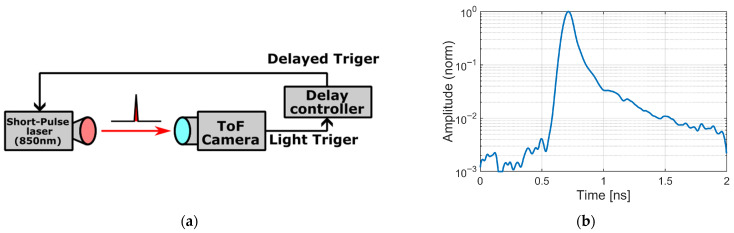
Impulse response measurement with picosecond laser pulses. (**a**) Measurement setup: 850 nm picosecond pulser (FWHM 70 ps) with a delay controller; the ToF camera provides the trigger. (**b**) Measured laser pulse waveform acquired with a photodetector and an oscilloscope (relative intensity, peak-normalized), used in subsequent Wiener deconvolution.

Figure 8a illustrates the measurement setup, in which the 850 nm laser directly illuminates the ToF camera, and Figure 8b shows the measured laser pulse waveform used in subsequent Wiener deconvolution [25]. Let $L\left(f\right)\;$ and $Y\left(f\right)$ denote the Fourier transforms of the measured laser waveform and the pixel output, respectively. The estimated impulse-response spectrum $H\left(f\right)$ was obtained by applying a Wiener filter,

(1) $$H\left(f\right)=\frac{L^{*}\left(f\right)Y\left(f\right)}{{\left|L\left(f\right)\right|}^{2}+NSR}$$

where * denotes complex conjugation and NSR is the noise-to-signal power ratio. In this work, a fixed value of $NSR\;=\;1.0\times 10^{-3}$ was used for all taps and for both the FSI and BSI sensors. According to the specified temporal response of the DX30BF, no significant tail is expected from the detector itself, so the small tail observed in the measured waveform is attributed to the temporal characteristics of the picosecond laser source.

**Figure 9:**
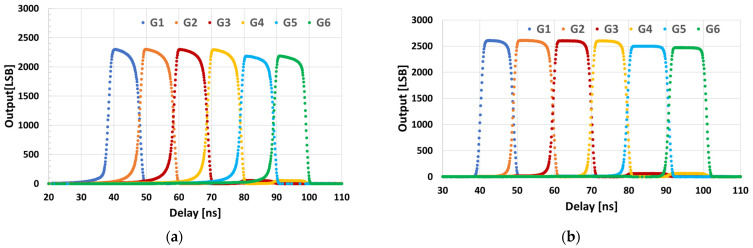
IRF measurement results with a gate-on time of 10 ns. (**a**) FSI sensor: per-tap outputs versus optical delay. (**b**) BSI sensor: flatter plateaus and steeper transitions than the FSI sensor, approaching an ideal 10 ns rectangular response.

Figure 9 shows the measured per-tap outputs for FSI and BSI when using the IRF protocol. The optical delay is swept with a fixed gate-on time of 10 ns. Six distinct windows corresponding to G1–G6 are evident in both devices. Compared to FSI, BSI exhibits flatter plateaus and steeper transitions, resulting in waveforms that more closely resemble an ideal 10 ns rectangular response.

**Figure 10:**
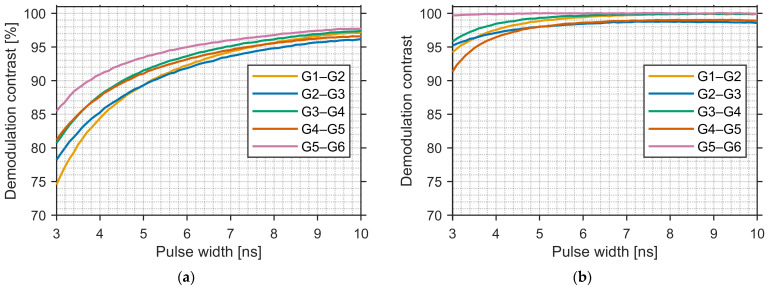
DC versus pulse width $T_{PW}$ for adjacent gate pairs. Values for $T_{PW}$ < 10 ns are estimated from the 10 ns data by a $\Delta T\;=\;10\;-\;T_{PW}$ ns time shift. (**a**) FSI sensor. (**b**) BSI sensor. BSI shows higher average DC than FSI (10 ns: 99.5% vs. 97.0%; 3 ns: 95.3% vs. 80.0%).

DC was evaluated from the sensor outputs in Figure 9 with a 10 ns gate. For each adjacent gate pair (G1–G2, G2–G3, G3–G4, G4–G5, G5–G6), DC was defined as the maximum value of $\left(G_{n}-G_{n+1}\right)/\left(G_{n}+G_{n+1}\right)$ observed during the sweep. Using this definition, DC was first computed at a gate width of 10 ns, and DC for shorter gate widths was estimated by evaluating the same ratio at a time shifted by $\Delta T\;=\;(10\;-\;T_{PW})$ ns toward the later side of the sweep; for example, the value at $T_{PW}$ is 3 ns was taken 7 ns later than the 10 ns optimum. Figure 10 summarizes DC versus pulse width $T_{PW}$ (3–10 ns) for all adjacent pairs. At $T_{PW}$ is 10 ns, average DC was 97.0% for FSI and 99.5% for BSI. At $T_{PW}$ is 3 ns, average DC was 80.0% for FSI and 95.3% for BSI, indicating reduced late-arriving components and enhanced temporal selectivity in the BSI device.

In the current setup, the pixel gates (G1–G6 and GD) are driven from different FPGA outputs via separate PCB traces. Small differences in propagation delay along these lines cause tap-dependent variations in the FWHM of the impulse responses, as seen in Figure 7. In particular, DC tends to be lower for tap pairs that include a tap with a slightly smaller FWHM. These variations, caused by timing skew in the external driver circuitry, are expected to be minimized by on-chip clock-distribution and synchronization techniques that align the gate timings across taps.

**Figure 11:**
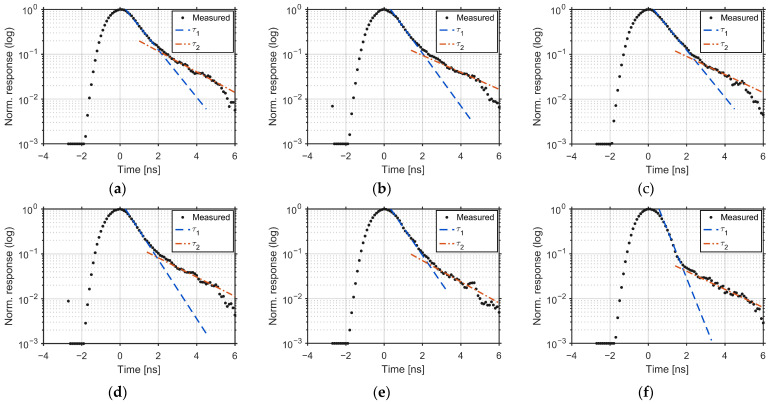
Per-tap IRFs for the FSI sensor: (**a**) G1, (**b**) G2, (**c**) G3, (**d**) G4, (**e**) G5, and (**f**) G6. The deconvolved waveforms have fitted time constants, $\tau_{1}$ (near-peak) and $\tau_{2}$ (tail), and the averages are taken across the taps. FWHM = 1.582 ns; $\tau_{1}$ = 0.692 ns; and $\tau_{2}$ = 2.058 ns.

Figure 11 presents the per-tap responses for the FSI sensor (G1–G6). In a lock-in pixel, the output of each tap corresponds to the time integral of the incident light within its time window. Therefore, the incident pulse waveform captured by each tap can be reconstructed by taking the derivative of the delay-swept output with respect to the gate delay. Each curve is the Wiener filtered response, fitted with a fast time constant τ_1_ near the peak and a slower time constant τ_2_ in the tail. The averages across taps are FWHM = 1.582 ns, *τ*_1_ = 0.692 ns, and *τ*_2_ = 2.058 ns.

**Figure 12:**
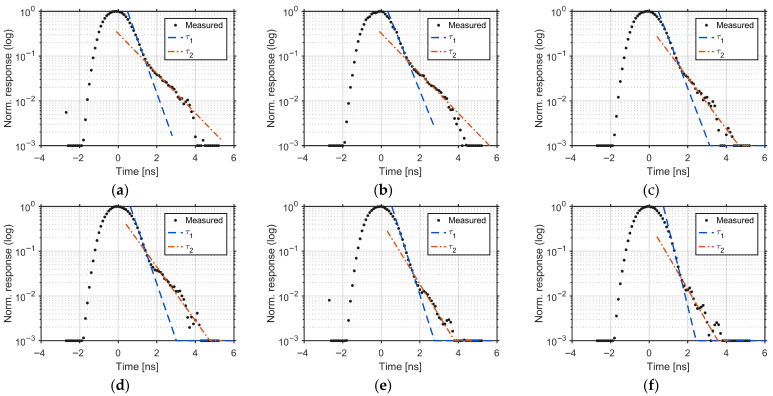
Per-tap IRFs for the BSI sensor: (**a**) G1, (**b**) G2, (**c**) G3, (**d**) G4, (**e**) G5, and (**f**) G6. Deconvolved waveforms with fitted *τ*_1_ and *τ*_2_; averages across taps: FWHM = 1.527 ns; $\tau_{1}$ = 0.345 ns; and $\tau_{2}$ = 0.774 ns.

Figure 12 shows the corresponding per-tap responses for the BSI sensor. The same fitting procedure is applied. The averages are FWHM = 1.527 ns, $\tau_{1}$ = 0.345 ns, and $\tau_{2}$ = 0.774 ns. The per-tap values for both devices are listed in Table 3. Both $\tau_{1}$ and $\tau_{2}$ are markedly smaller than those of FSI, indicating faster demodulation with reduced late-arriving charge. Compared to FSI, BSI reduces both time constants by approximately half on average ($\tau_{1}$: 0.692 ns → 0.345 ns; $\tau_{2}$: 2.058 ns → 0.774 ns) and also yields a modest reduction in full width at half maximum (FWHM) (1.582 ns → 1.527 ns), indicating a narrower impulse response. These improvements are consistent with faster charge transfer and reduced late-arriving charge in the BSI structure.

In this interpretation, $\tau_{1}$ represents the fast, dominant component of photoelectron transfer from regions close to the transfer gates, whereas $\tau_{2}$ corresponds to a slower tail caused by late-arriving charge. In the BSI sensor, thinning the substrate suppresses the contribution of carriers generated deep in the silicon, but the remaining $\tau_{2}$ suggests that carriers generated farther from the demodulation gates (G1–G6) in the lateral direction, such as near the photodiode edges, still require more time to reach the demodulation region.

For reference, the time constants calculated from sensor output without Wiener filtering (tap-wise averages) were 0.93 ns ($\tau_{1}$) and 2.91 ns ($\tau_{2}$) for the FSI device, and 0.46 ns ($\tau_{1}$) and 0.85 ns ($\tau_{2}$) for the BSI device [21]. After applying Wiener deconvolution (Figure 8 and Figure 9), these constants decreased by 25.6% ($\tau_{1}$) and 29.3% ($\tau_{2}$) in FSI, and by 25.0% ($\tau_{1}$) and 8.9% ($\tau_{2}$) in BSI, revealing the intrinsic IRF.

In the BSI structure, introducing a reflector enables the sensor to achieve QE at 940 nm comparable to that of an FSI pixel with a 20 µm-thick silicon epitaxial layer (20.6% vs. 19.7%), even though the active silicon layer is thinned. Since the active silicon layer is thinner in the BSI sensor, the time constants $\tau_{1}$ and $\tau_{2}$ are approximately two to three times shorter than those in the FSI pixel, indicating a much faster response. Therefore, the BSI structure with the reflector improves pixel speed by a factor of about two to three while achieving high NIR QE comparable to that of the FSI pixel.

### 3.3. Range Measurement

Indoor range measurements were performed using a BSI SP-iToF camera with two subframes (SF1 and SF2). The light source was a pulsed 940 nm laser with a width of 15 ns and a period of 300 ns (5% duty cycle). A 940 ± 10 nm band-pass filter was mounted on the camera. The frame rate was 30 frames per second, and planar targets were placed at distances ranging from 3 to 28 m. The measurement conditions are summarized in Table 4.

**Figure 13:**
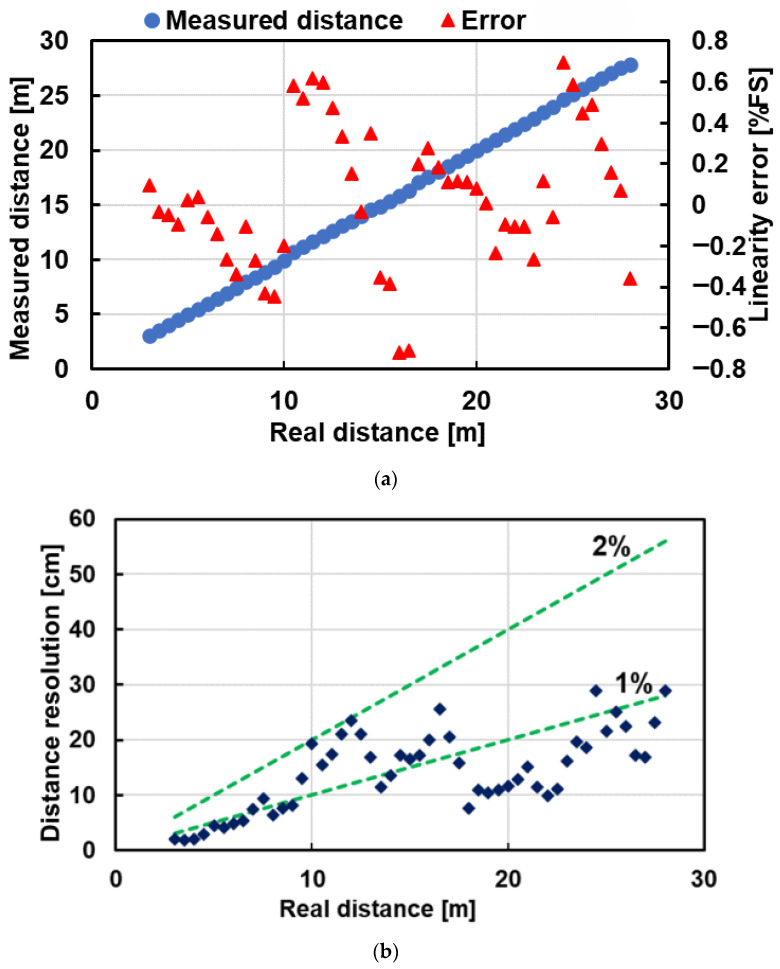
Range measurement results: (**a**) Measured distance (left axis) and linearity error (right axis). (**b**) Distance resolution versus distance. The error remains within ±0.8%FS over the range of 3–28 m, and the resolution stays below 2%.

**Figure 14:**
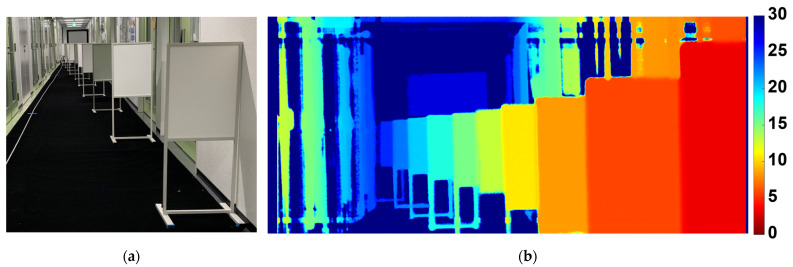
The measurement scene and an example distance image were captured by the BSI six-tap iToF camera. (**a**) Indoor measurement setup and (**b**) reconstructed distance image.

Figure 13a plots measured distance versus ground-truth distance together with the linearity error (right axis, %FS). The error remains within ±0.8%FS over 3–28 m. Figure 13b shows the distance resolution versus distance. The resolution stays below 2% across the whole range and falls below 1% at several distances. Figure 14 shows the measurement scene and a distance image taken with the BSI six-tap iToF camera.

Under these conditions, the six-tap BSI sensor measures ranges from 3 to 28 m at 30 fps. In a BSI four-tap sensor [12], ranges from 1 to 20 m were measured at a frame rate of 15 fps. This comparison demonstrates that increasing the number of taps allows a wider measurable range to be covered with fewer subframes, which in turn makes it possible to achieve a higher frame rate at similar or larger ranges. In general, there is a clear trade-off between the number of subframes (and thus the measurable ToF range) and the achievable frame rate: using more subframes extends the range, but the frame rate decreases. By using more taps, the required number of subframes can be kept small while still covering a wide range. Even when the tap count is increased, only minor adjustments to the gate-timing pattern are needed, so the overall system architecture does not require major changes. In this sense, increasing the tap count in a BSI multi-tap pixel is an effective way to relax the range–frame-rate trade-off while keeping the system complexity low.

## 4. Conclusions

Using an identical pixel layout, we experimentally demonstrate that a backside-illuminated (BSI) six-tap iToF sensor exhibits faster demodulation performance than its front-side illuminated (FSI) counterpart. Per-tap impulse response measurements reveal that the BSI time constants—*τ*_1_ (near-peak) and *τ*_2_ (tail)—are each less than half those observed in the FSI structure. Device simulations support a carrier transport mechanism, wherein reducing the initial electron generation depth from 10 μm to 5 μm shortens the transfer time and narrows the carrier arrival-time distribution. The average demodulation contrast (DC) of the BSI device reached 99.5% at a gate-on time of 10 ns, outperforming the FSI device (97.0%). When the gate-on time was reduced to 3 ns, the contrast margin widened further (95.3% vs. 80.0%). This enhanced DC, particularly at shorter pulse widths, enables the realization of ToF sensors with exceptional precision and linearity.

Since ToF precision is directly proportional to the gating pulse width—which matches the optical pulse width [26]—future work will focus on demonstrating high-precision, high-linearity ToF measurements at short ranges using optical pulses of 5 ns or less, while maintaining high demodulation contrast in short-range applications such as motion capture and factory automation. Operation with sub-5 ns gating pulses makes the system more sensitive to pulse-shape distortion and noise, so high-speed, low-distortion drivers for the optical source and the demodulation gates will be required. We also plan to quantify pixel-to-pixel and frame-to-frame variations in demodulation contrast, time constants, and range accuracy, and to investigate power, thermal, and manufacturing trade-offs of the BSI structure, including outdoor and high-ambient-light tests, to clarify the long-term stability and practical suitability of BSI six-tap iToF sensors.

## Figures and Tables

**Table 1:** Simulated charge-transfer time Δt [ps] to FD for each gate asserted High (other gates held Low). Initial starting depths are 5 and 10 µm.

Gate (High)	Δt [ps]	
	5 µm	10 µm
G1	154	265
G3	156	267
G5	163	274
Drain (GD)	141	252
Average	153.5	264.5

**Table 2:** Simulated carrier-transfer time Δt [ps] from each starting point P(x, y, z) (red circles in Figure 4a) to gate GD in the FSI pixel. The initial depths in the p-epitaxial layer are z = 5 µm and z = 10 µm (GD High, G1–G6 Low).

z [µm]	y [µm]	Δt [ps]				
		x = 1.4 [µm]	x = 2.4 [µm]	x = 3.4 [µm]	x = 4.4 [µm]	x = 5.4 [µm]
5	2.2	1160	1120	206	169	150
	4.2	377	307	141	125	107
	6.2	1160	1120	206	169	150
10	2.2	1271	1230	318	280	261
	4.2	490	419	252	236	218
	6.2	1271	1230	318	280	261

**Table 3:** Summary of per-tap parameters extracted from the IRFs for FSI and BSI.

Gate	FSI			BSI		
	FWHM [ns]	*τ*_1_	*τ*_2_	FWHM [ns]	*τ*_1_	*τ*_2_
G1	1.628	0.823	1.890	1.522	0.362	0.977
G2	1.598	0.739	2.294	1.465	0.408	0.973
G3	1.639	0.827	2.154	1.559	0.386	0.757
G4	1.547	0.661	2.025	1.589	0.346	0.726
G5	1.590	0.697	1.837	1.517	0.320	0.620
G6	1.487	0.407	2.150	1.510	0.246	0.590
Average	1.582	0.692	2.058	1.527	0.345	0.774

**Table 4:** Measurement conditions for the range measurement test.

Measurement Parameter	Setting
Chip process	0.11-µm CIS (BSI)
Number of Subframes	2
Wavelength of LD	940 nm
NIR band filter	940 ± 10 nm
Light pulse width	15 ns
Duty ratio of light pulse	5%
Frame rate	30 fps
Environment	Indoor
Distance range	3–28 m

## Data Availability

The original contributions presented in this study are included in the article. Further inquiries can be directed to the corresponding author.
